# Supplementary material for: A Holistic Evolutionary and 3D Pharmacophore Modelling Study Provides Insights into the Metabolism, Function, and Substrate Selectivity of the Human Monocarboxylate Transporter 4 (hMCT4)
Source: Int J Mol Sci. 2021 Mar 13;22(6):2918. doi: 10.3390/ijms22062918 (PMC8001682; doi:10.3390/ijms22062918)
Supplement: Supplementary file 1 [file ijms-22-02918-s001.pdf]

Table S1. Energy calculations from the docking simulations.

hMCT4 model	Eint (kcal/mol)	ratio
**Syrosingopine**	-120.303	1
**AR-C155858**	-85.244	0.709
**CHC**	-37.096	0.308
**Phloretin**	-87.867	0.730
**Quercetin**	-37.508	0.312
**AZD3965**	-65.723	0.546
**7ACC2**	-54.826	0.456
**BNG**	-39.970	0.332
hGLUT1 (4PYP)	**Eint (kcal/mol)**	
**BNG**	-77.805	

Docking simulation were performed for the seven compounds against the human MCT4 model and their interaction energies were calculated after minimization. Since the most relative human 3-D structure for a transporter co-crystallized with an inhibitor is the human glucose transporter GLUT1 with nonyl beta-D-glucopyranoside (BNG) (PDB ID: 4PYP), its interaction energy was also calculated. Consequently, BNG was docked against the hMCT4 model to evaluate its interaction energy, that is comparably larger than all calculated energies. Syrosingopine exerts the lowest interaction energy and a column of energy ratios ($E_i/E_{Syrosingopine}$) added in the table shows the relative differences.

Table S2. Toxicity information of the MCT inhibitors.

Compound		
**Syrosingopine**	Syrosingopine is a known antihypertensive agent derived from reserpine that was found to potentiate the anticancer effects of metformin and phenformin without harmful effects on normal cells.	[S1]
**AR-C155858**	AR-C155858 is a potent MCT-1 inhibitor, that showed no evident toxicity in an animal model study.	[S2]
**CHC**	CHC administration showed no signs of morbidity in an animal study with a 30-day toxicity trial.	[S3]
**Phloretin**	Phloretin is an antioxidant polyphenol, that exerts dose-dependent toxicity.	[S4]
**Quercetin**	Quercetin is a flavonoid with antioxidant and anti-inflammatory effects, with a safe clinical profile as demonstrated in a Phase I Clinical Trial (NCT01708278).	[S5]
**AZD3965**	AZD3965 is an MCT1 inhibitor safe for clinical use, and is currently in a Phase I Clinical Trial (NCT01791595).	[S6, S7]
**7ACC2**	7ACC2 is a specific MCT1 inhibitor showing no toxicity in cells using glucose instead of lactate and is anticipated to have a safe profile.	[S8, S9]

Table S3. List of aminoacid sequences included in the phylogenetic tree of this study (Figure 1, Figure S1).

A/A	RefSeq[1] code	Species
1	NP_001139004	Equus caballus
2	NP_001035887	Homo sapiens
3	NP_997873	Danio rerio
4	NP_001007912	Xenopus tropicalis
5	NP_001033743	Mus musculus
6	NP_001103450	Bos taurus
7	NP_989994	Gallus gallus
8	NP_001233224	Takifugu rubripes
9	NP_001125113	Pongo abelii
10	GAT30004	Aspergillus luchuensis
11	XP_010886933	Esox lucius
12	XP_005144268	Melopsittacus undulatus
13	XP_012390575	Orcinus orca
14	XP_002197467	Taeniopygia guttata
15	XP_015735135	Coturnix japonica
16	XP_015090880	Vicugna pacos
17	XP_003211508	Meleagris gallopavo
18	XP_006740558	Leptonychotes weddellii
19	XP_010352658	Rhinopithecus roxellana
20	XP_004709270	Echinops telfairi
21	XP_008492797	Calypte anna
22	XP_009092511	Serinus canaria
23	XP_017928676	Manacus vitellinus
24	XP_008826319	Nannospalax galili
25	XP_015673014	Protobothrops mucrosquamatus
26	XP_010160653	Antrostomus carolinensis
27	XP_011195890	Zeugodacus cucurbitae
28	XP_007063427	Chelonia mydas
29	XP_005436437	Falco cherrug
30	XP_005243993	Falco peregrinus
31	XP_003496959	Cricetulus griseus
32	XP_005495722	Zonotrichia albicollis
33	XP_005350966	Microtus ochrogaster
34	XP_011250974	Camponotus floridanus
35	XP_006038950	Alligator sinensis
36	XP_007424641	Python bivittatus
37	XP_023950162	Bicyclus anynana
38	XP_011476964	Oryzias latipes
39	XP_013002653	Cavia porcellus
40	XP_003786090	Otolemur garnettii
41	XP_023300723	Lucilia cuprina
42	XP_014205912	Copidosoma floridanum

43	XP_022231531	Drosophila obscura
44	XP_022114180	Pieris rapae
45	XP_005069842	Mesocricetus auratus
46	XP_012632713	Microcebus murinus
47	GAQ45312	Aspergillus niger
48	KQK81225	Amazona aestiva
49	GAA92712	Aspergillus kawachii IFO
50	EPY74356	Camelus ferus
51	XP_026771377	Pangasianodon hypophthalmus
52	XP_026171558	Mastacembelus armatus
53	XP_023049889	Piliocolobus tephrosceles
54	XP_020316761	Oncorhynchus kisutch
55	XP_021389729	Lonchura striata domestica
56	XP_026510410	Terrapene carolina triunguis
57	XP_021069026	Mus pahari
58	XP_018617229	Scleropages formosus
59	XP_028990271	Betta splendens
60	XP_028912882	Ornithorhynchus anatinus
61	XP_014976124	Macaca mulatta
62	XP_028841183	Denticeps clupeoides
63	XP_028674226	Erpetoichthys calabaricus
64	XP_028572122	Podarcis muralis
65	XP_028643717	Grammomys surdaster
66	XP_020901285	Exaiptasia pallida
67	XP_028375965	Phyllostomus discolor
68	XP_028332832	Gouania willdenowi
69	XP_028023154	Balaenoptera acutorostrata scammoni
70	XP_008153193	Eptesicus fuscus
71	XP_027879094	Xiphophorus couchianus
72	XP_027968245	Eumetopias jubatus
73	XP_027480584	Zalophus californianus
74	XP_026870538	Electrophorus electricus
75	XP_026716986	Athene cunicularia
76	XP_026557668	Pseudonaja textilis
77	XP_026266521	Urocitellus parryii
78	XP_025988236	Solenopsis invicta
79	XP_025926974	Apteryx rowi
80	XP_025904910	Nothoprocta perdicaria
81	XP_025855772	Vulpes vulpes
82	XP_025783425	Puma concolor
83	XP_025762468	Oreochromis niloticus
84	XP_025715506	Callorhinus ursinus
85	XP_025564430	Aspergillus vadensi
86	XP_025540281	Aspergillus costaricaensis
87	XP_025513713	Aspergillus piperis CBS
88	XP_025480558	Aspergillus neoniger

89	XP_025324183	Canis lupus dingo
90	XP_025135542	Bubalus bubalis
91	XP_025034368	Pelodiscus sinensis
92	XP_024781965	Pan paniscus
93	XP_024659285	Maylandia zebra
94	XP_024409579	Desmodus rotundus
95	XP_024128461	Oryzias melastigma
96	XP_023960498	Chrysemys picta bellii
97	XP_006108461	Myotis lucifugus
98	XP_023584961	Trichechus manatus latirostris
99	XP_023555060	Octodon degus
100	XP_023446052	Dasypus novemcinctus
101	XP_023205320	Xiphophorus maculatus
102	PKU32899	Limosa lapponica baueri
103	XP_022621787	Seriola dumerili
104	XP_022524772	Astyanax mexicanus
105	XP_022378873	Enhydra lutris kenyoni
106	XP_022278316	Canis lupus familiaris
107	XP_021586982	Ictidomys tridecemlineatus
108	XP_021564173	Carlito syrichta
109	XP_021536557	Neomonachus schauinslandi
110	XP_021493108	Meriones unguiculatus
111	XP_021431909	Oncorhynchus mykiss
112	XP_021270295	Numida meleagris
113	XP_021163211	Fundulus heteroclitus
114	XP_005515822	Columba livia
115	XP_021096896	Heterocephalus glaber
116	XP_020922027	Sus scrofa
117	XP_020856620	Phascolarctos cinereus
118	XP_020762975	Odocoileus virginianus texanus
119	XP_020670380	Pogona vitticeps
120	XP_020464014	Monopterus albus
121	XP_020440499	Corvus cornix cornix
122	OPJ79552	Patagioenas fasciata monilis
123	JAV37409	Castor canadensis
124	KLP22203	Fusarium fujikuroi
125	JAB41924	Callithrix jacchus
126	BAL44664	Danio rerio
127	XP_034413768	Cyclopterus lumpus
128	XP_034362697	Arvicanthis niloticus
129	XP_034268834	Pantherophis guttatus
130	XP_034083463	Gymnodraco acuticeps
131	XP_034046144	Thalassophryne amazonica
132	XP_033969593	Trematomus bernacchii
133	XP_033945398	Pseudochaenichthys georgianus
134	XP_033826910	Periophthalmus magnuspinnatus

135	KAF4501961	Fusarium agapanthi
136	KAF4471748	Fusarium albosuccineum
137	KAF4444891	Fusarium acutatum
138	XP_033817369	Geotrypetes seraphini
139	KAF4345618	Fusarium beomiforme
140	XP_010616978	Fukomys damarensis
141	XP_033467119	Epinephelus lanceolatus
142	XP_026206432	Anabas testudineus
143	XP_033037571	Trachypithecus francoisi
144	XP_032995022	Lacerta agilis
145	XP_030362108	Strigops habroptila
146	XP_032932565	Catharus ustulatus
147	XP_032857772	Tyto alba alba
148	XP_032900471	Amblyraja radiata
149	XP_032769452	Rattus rattus
150	XP_032726224	Lontra canadensis
151	XP_032034294	Hylobates moloch
152	XP_032443028	Xiphophorus hellerii
153	XP_032358773	Etheostoma spectabile
154	XP_032285323	Phoca vitulina
155	XP_032225716	Nematostella vectensis
156	XP_032097112	Sapajus apella
157	XP_032066854	Thamnophis elegans
158	XP_031984563	Corvus moneduloides
159	XP_031729757	Anarrhichthys ocellatus
160	XP_031454898	Phasianus colchicus
161	XP_031325277	Camelus dromedarius
162	XP_031210787	Mastomys coucha
163	XP_005429851	Geospiza fortis
164	XP_030853498	Strongylocentrotus purpuratus
165	XP_030818215	Camarhynchus parvulus
166	XP_030390726	Gopherus evgoodei
167	XP_030373139	Scaptodrosophila lebanonensis
168	XP_030196761	Gadus morhua
169	XP_030061057	Microcaecilia unicolor
170	XP_029828573	Ixodes scapularis
171	XP_029870524	Aquila chrysaetos chrysaetos
172	XP_029784684	Suricata suricatta
173	XP_029620865	Salmo trutta
174	XP_029541028	Oncorhynchus nerka
175	XP_012541288	Monomorium pharaonis
176	RZR69168	Pochonia chlamydosporia
177	XP_027776423	Marmota flaviventris
178	XP_027728942	Vombatus ursinus
179	XP_027745586	Empidonax traillii
180	XP_027573086	Pipra filicauda

181	XP_027525769	Corapipo altera
182	XP_027373650	Bos indicus x Bos taurus
183	XP_027327340	Anas platyrhynchos
184	XP_010749368	Larimichthys crocea
185	XP_027033331	Tachysurus fulvidraco
186	RMZ69625	Pyrenophora seminiperda
187	XP_026523140	Notechis scutatus
188	RGP75237	Fusarium sporotrichioides
189	RGP65035	Fusarium longipes
190	RFN45264	Fusarium fasciculatum
191	XP_017294327	Kryptolebias marmoratus
192	XP_023678435	Paramormyrops kingsleyae
193	XP_012157214	Ceratitis capitata
194	XP_002075246	Drosophila willistoni
195	XP_003176299	Nannizzia gypsea CBS
196	KPA46312	Fusarium langsethiae

[1]The NCBI Reference Sequence (RefSeq) provides sequence records and related information for numerous organisms, and provides a baseline for medical, functional, and comparative studies. Whereas the International Nucleotide Sequence Database Collaboration (INSDC, made up of GenBank, the European Nucleotide Archive, and the DNA Data Bank of Japan) represents an archival repository of all sequences, the RefSeq database is a non-redundant set of reference standards derived from the INSDC databases that includes chromosomes, complete genomic molecules (organelle genomes, viruses, plasmids), intermediate assembled genomic contigs, curated genomic regions, mRNAs, RNAs, and proteins. Accession numbers that begin with the prefix XM_ (mRNA), XR_ (non-coding RNA), and XP_ (protein) are model RefSeqs produced either by NCBI's genome annotation pipeline or copied from computationally annotated submissions to the INSDC. These RefSeq records are derived from the genome sequence and have varying levels of transcript or protein homology support. They represent the predicted transcripts and proteins annotated on the NCBI RefSeq contigs and may differ from INSDC mRNA submissions or from the subsequently curated RefSeq records (with NM_, NR_, or NP_ accession prefixes). These differences may reflect real sequence variation (polymorphism), or errors or gaps in the available genome sequence. (www.ncbi.nlm.nih.gov/books/NBK21091/)

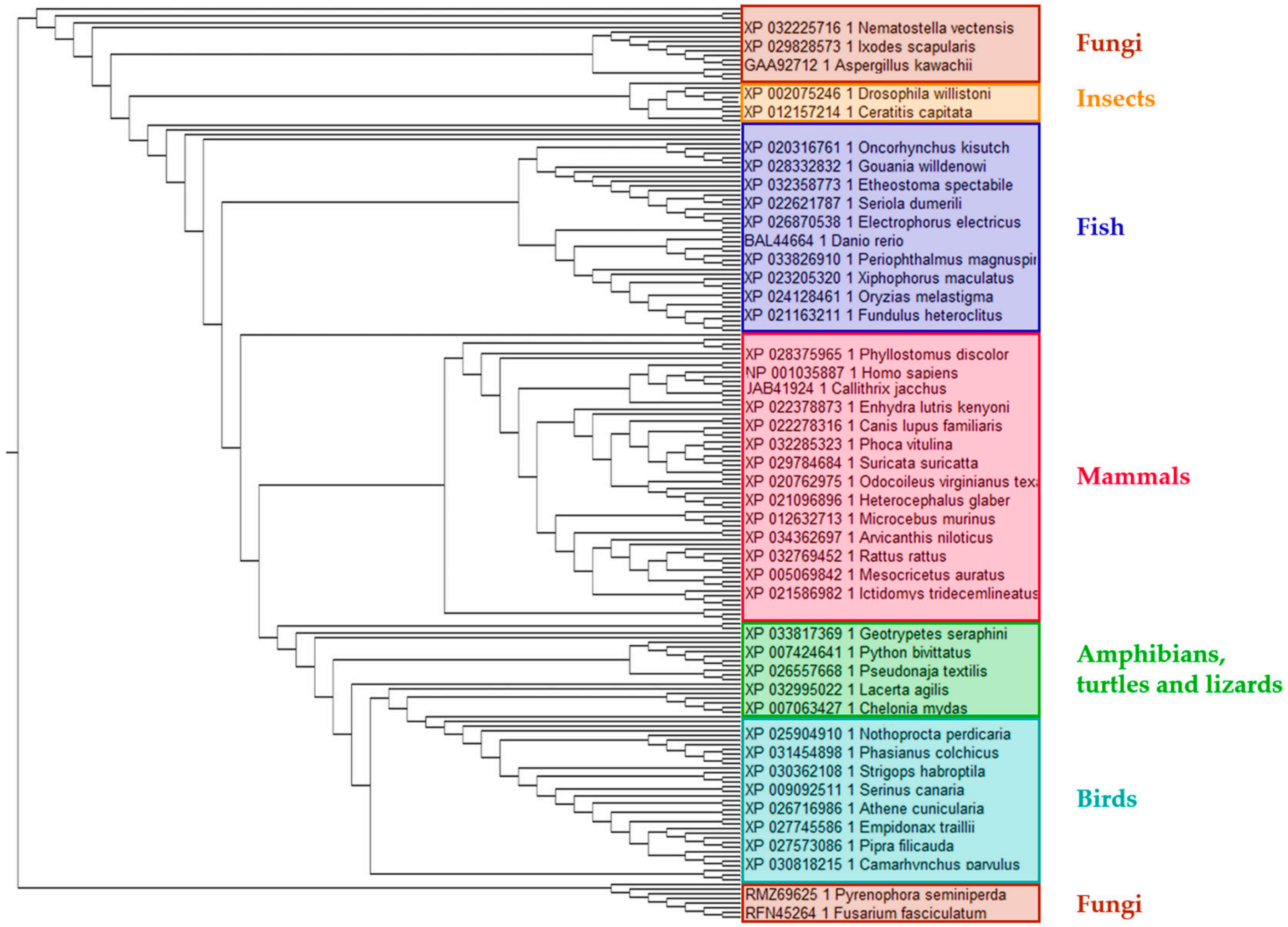

Figure S1: The phylogenetic tree of the MCT4 across species (shown in Figure1) in vertical form with annotated representative species.

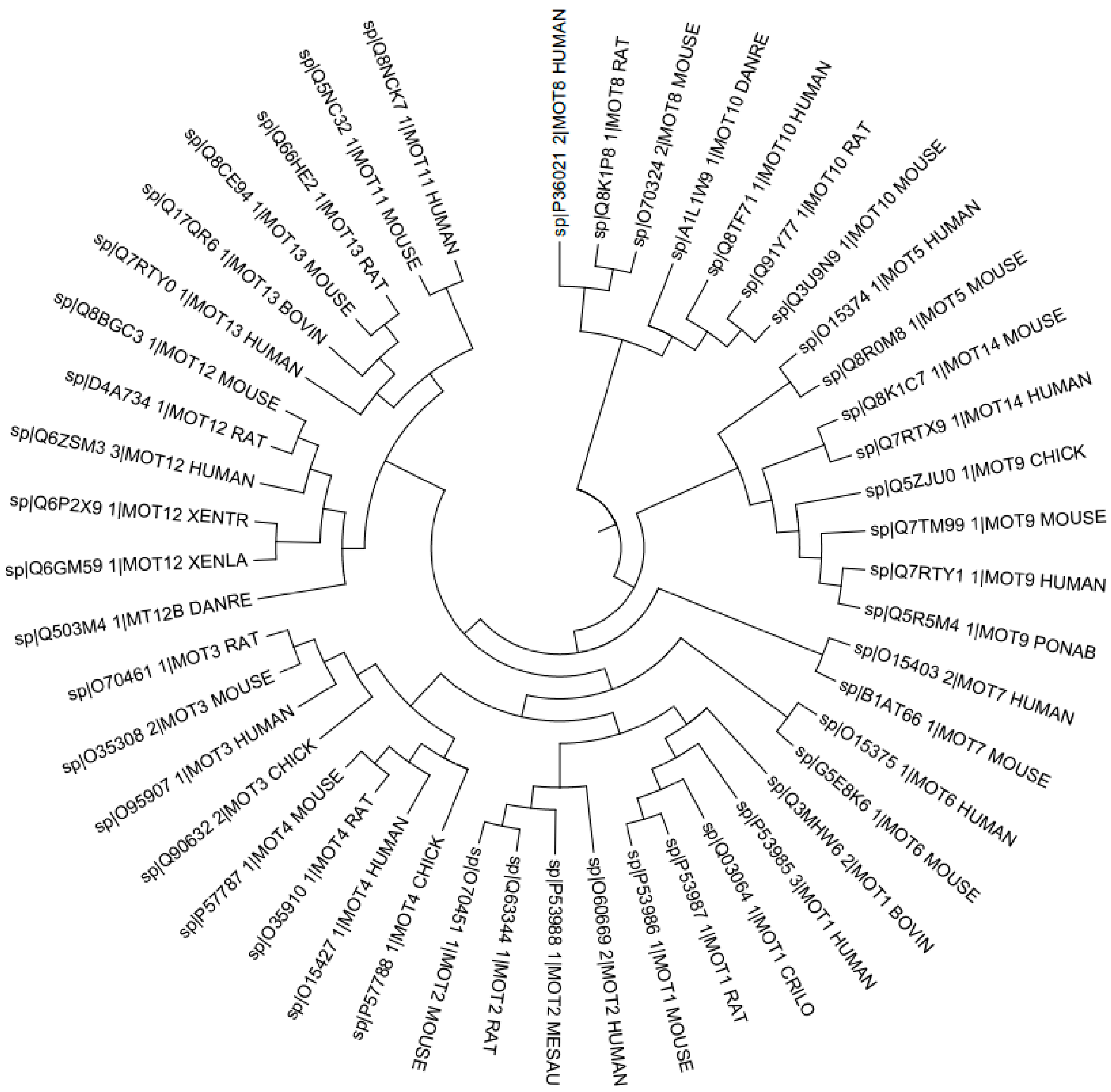

Figure S2. Phylogenetic tree of the SLC16 members in mammals

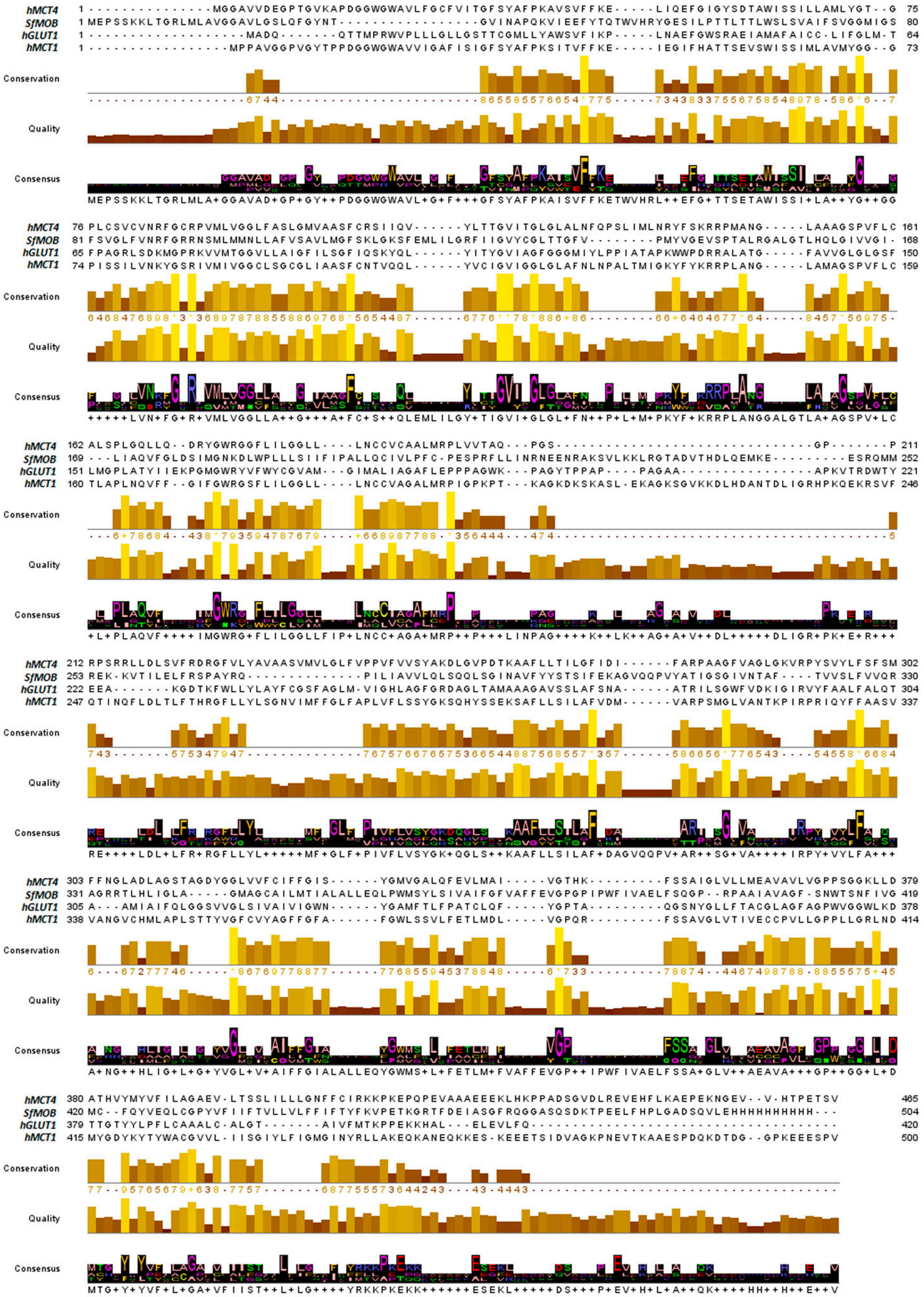
hMCT4
SfMOB
hGLUT1
hMCT1
Conservation
Quality
Consensus

B

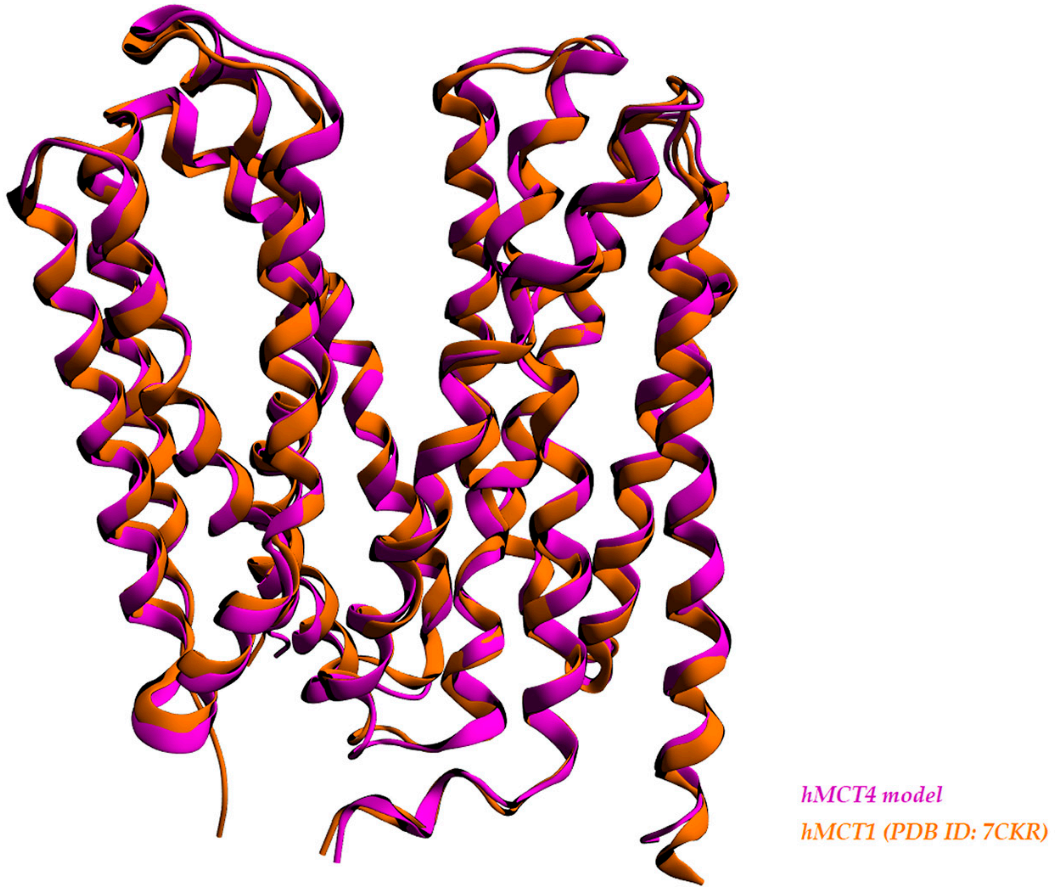

Figure S3. (A) Sequence alignment of hMCT4, 6G9X, 4PYP and.hMCT1. (B) Structural superposition of the backbone of hMCT4 and hMCT1 (RMSD = 0.936Å).

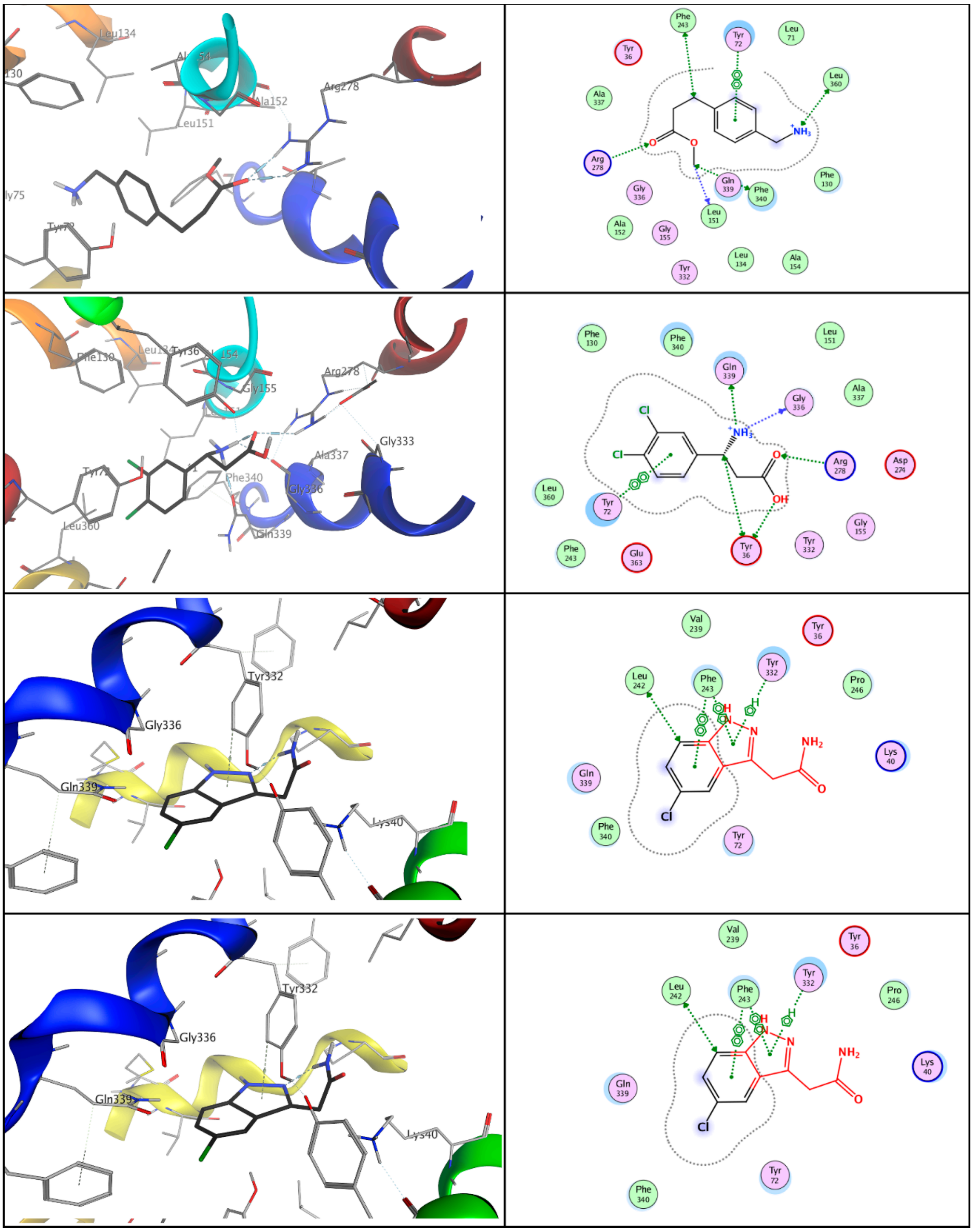

Figure S4: Potent inhibitors of hMCT4 through a pharmacophore-based high throughput screening. A high throughput virtual screening of small molecule and anti-cancer chemical databases was performed using the MOE platform against the designed pharmacophore. Four of the top hits (compound 1-4) are presented in their docking position (left) along with the ligand-protein interactions (right).

References

S1. Benjamin, D.; Colombi, M.; Hindupur, S.K.; Betz, C.; Lane, H.A.; El-Shemerly, M.Y.; Lu, M.; Quagliata, L.; Terracciano, L.; Moes, S., et al. Syrosingopine sensitizes cancer cells to killing by metformin. Sci Adv 2016, 2, e1601756, doi:10.1126/sciadv.1601756.

S2. Pahlman, C.; Qi, Z.; Murray, C.M.; Ferguson, D.; Bundick, R.V.; Donald, D.K.; Ekberg, H. Immunosuppressive properties of a series of novel inhibitors of the monocarboxylate transporter MCT-1. Transpl Int 2013, 26, 22-29, doi:10.1111/j.1432-2277.2012.01579.x.

S3. Kim, H.S.; Masko, E.M.; Poulton, S.L.; Kennedy, K.M.; Pizzo, S.V.; Dewhirst, M.W.; Freedland, S.J. Carbohydrate restriction and lactate transporter inhibition in a mouse xenograft model of human prostate cancer. BJU Int 2012, 110, 1062-1069, doi:10.1111/j.1464-410X.2012.10971.x.

S4. Geohagen, B.C.; Korsharskyy, B.; Vydyanatha, A.; Nordstroem, L.; LoPachin, R.M. Phloretin cytoprotection and toxicity. Chem Biol Interact 2018, 296, 117-123, doi:10.1016/j.cbi.2018.09.020.

S5. Han, M.K.; Barreto, T.A.; Martinez, F.J.; Comstock, A.T.; Sajjan, U.S. Randomised clinical trial to determine the safety of quercetin supplementation in patients with chronic obstructive pulmonary disease. BMJ Open Respir Res 2020, 7, doi:10.1136/bmjresp-2018-000392.

S6. Allen, A.E.; Martin, E.A.; Greenwood, K.; Grant, C.; Vince, P.; Lucas, R.J.; Redfern, W.S. Effects of a monocarboxylate transport 1 inhibitor, AZD3965, on retinal and visual function in the rat. Br J Pharmacol 2020, 177, 4734-4749, doi:10.1111/bph.15239.

S7. McNeillis, R.; Greystoke, A.; Walton, J.; Bacon, C.; Keun, H.; Siskos, A.; Petrides, G.; Leech, N.; Jenkinson, F.; Bowron, A., et al. A case of malignant hyperlactaemic acidosis appearing upon treatment with the mono-carboxylase transporter 1 inhibitor AZD3965. Br J Cancer 2020, 122, 1141-1145, doi:10.1038/s41416-020-0727-8.

S8. Draoui, N.; Schicke, O.; Fernandes, A.; Drozak, X.; Nahra, F.; Dumont, A.; Douxfils, J.; Hermans, E.; Dogne, J.M.; Corbau, R., et al. Synthesis and pharmacological evaluation of carboxycoumarins as a new antitumor treatment targeting lactate transport in cancer cells. Bioorg Med Chem 2013, 21, 7107-7117, doi:10.1016/j.bmc.2013.09.010.

S9. Draoui, N.; Schicke, O.; Seront, E.; Bouzin, C.; Sonveaux, P.; Riant, O.; Feron, O. Antitumor activity of 7-aminocarboxycoumarin derivatives, a new class of potent inhibitors of lactate influx but not efflux. Mol Cancer Ther 2014, 13, 1410-1418, doi:10.1158/1535-7163.MCT-13-0653.
